# Supplementary material for: The Systems Biology Research Tool: evolvable open-source software
Source: BMC Syst Biol. 2008 Jun 29;2:55. doi: 10.1186/1752-0509-2-55 (PMC2446383; doi:10.1186/1752-0509-2-55)
Supplement: Additional file 1 — SBRT Archive. An archive of the current version of the Systems Biology Research Tool. [file 1752-0509-2-55-S1.zip › sbrt-1.4.0/doc/developers_guide/api/sbrt/kernel/math/geom/comp/CdHarComputer.html]

CdHarComputer


|  |  |  |
| --- | --- | --- |
| **Overview** \| **Package** \| **Class** \| **Use** \| **Tree** \| **Deprecated** \| **Index** \| **Help** | |  |
| PREV CLASS   **NEXT CLASS** | **FRAMES**    **NO FRAMES**     **All Classes** |  |
| SUMMARY: NESTED \| FIELD \| CONSTR \| METHOD | DETAIL: FIELD \| CONSTR \| METHOD |  |


---


## sbrt.kernel.math.geom.comp Class CdHarComputer<V>

```
java.lang.Object
  sbrt.kernel.math.geom.comp.CdHarComputer<V>
```

**Type Parameters:**: `V` - the variable type.

---

``` public class CdHarComputer<V> extends java.lang.Object ```

This class is an implemenation of a coordinate-direction hit-and-run
algorithm.

**Author:**
:   This class was written and documented by Jeremiah Wright while in the
    Wagner lab.

---

| **Nested Class Summary** | |
| --- | --- |
| `class` | `CdHarComputer.Results`             This class is used to represent the results of a random point computation. |


**Constructor Summary**

`CdHarComputer(ConvexPolytope<V> polytope)`
  
          Constructs a new random point generator for the provided convex polytope.


| **Method Summary** | |
| --- | --- |
| `double` | `getConstraintTolerance()`             Returns the constraint tolerance. |
| `double` | `getMinChordLength()`             Returns the minimum length a computed chord must have to be considered successful. |
| `ConvexPolytope<V>` | `getPolytope()`             Returns the convex polytope for which random points are generated. |
| `CdHarComputer.Results` | `next()`             Computes and returns the next random interior point of the convex polytope. |
| `CdHarComputer.Results` | `next(int totalPoints)`             Computes the specified number of random interior points, and returns the last one computed. |
| `CdHarComputer.Results` | `run(java.util.Map<? extends V,java.lang.Double> startPoint)`             Computes and returns a random interior point of the convex polytope starting from the provided point. |
| `void` | `setConstraintTolerance(double tolerance)`             Sets the constraint tolerance. |
| `void` | `setMinChordLength(double minChordLength)`             Sets the minimum chord length. |
| `void` | `setPoint(java.util.Map<? extends V,java.lang.Double> startPoint)`             Sets the initial point. |
| `void` | `setRandom(java.util.Random random)`             Sets the random number generator. |

**Methods inherited from class java.lang.Object**

`clone, equals, finalize, getClass, hashCode, notify, notifyAll, toString, wait, wait, wait`

**Constructor Detail**

### CdHarComputer

```
public CdHarComputer(ConvexPolytope<V> polytope)
```

:   Constructs a new random point generator for the provided convex polytope.

    **Parameters:**: `polytope` - the convex polytope for which random interior points are to be generated. **Throws:**: `DimensionException` - if the dimension of the provided polytope is < 1.


**Method Detail**

### setPoint

```
public void setPoint(java.util.Map<? extends V,java.lang.Double> startPoint)
```

:   Sets the initial point. This method sets the initial interior point from
    which the algorithm is started.

    :   **Parameters:**: `startPoint` - variables mapped to their numerical values. **Throws:**: `InvalidPointException` - if the provided point does not lie within the polytope.

---


### setRandom

```
public void setRandom(java.util.Random random)
```

:   Sets the random number generator.

    :   **Parameters:**: `random` - the random number generator with which all psuedorandom numbers are to be produced.

---


### setConstraintTolerance

```
public void setConstraintTolerance(double tolerance)
```

:   Sets the constraint tolerance. This is the amount by which constraints
    can be violated.

    :   **Parameters:**: `tolerance` - the amount by which constaints can be violated. This should be a small positive number, like 1E-9 for example.

---


### setMinChordLength

```
public void setMinChordLength(double minChordLength)
```

:   Sets the minimum chord length. If a computed chord is shorter than the
    provided amount, the move is considered obstructed. This can happen if the
    random walker gets stuck in a corner.

    :   **Parameters:**: `minChordLength` - the minimum length a chord must have to be considered valid. This should be a small positive number, like 1E-9 for example.

---


### getConstraintTolerance

```
public double getConstraintTolerance()
```

:   Returns the constraint tolerance. This is the amount by which constraints
    can be violated.

    :   **Returns:**: the constraint tolerance. **See Also:**: `setConstraintTolerance(double)`

---


### getMinChordLength

```
public double getMinChordLength()
```

:   Returns the minimum length a computed chord must have to be considered
    successful. Chords with a length smaller than the returned value are
    considered obstructed moves.

    :   **Returns:**: the minimum chord length. **See Also:**: `setMinChordLength(double)`

---


### getPolytope

```
public ConvexPolytope<V> getPolytope()
```

:   Returns the convex polytope for which random points are generated.

    :   **Returns:**: the convex polytope for which random points are generated.

---


### next

```
public CdHarComputer.Results next()
```

:   Computes and returns the next random interior point of the convex
    polytope.

    :   **Returns:**: the next random interior point of the convex polytope.

---


### next

```
public CdHarComputer.Results next(int totalPoints)
```

:   Computes the specified number of random interior points, and returns the
    last one computed. The changed and unchanged variables in the returned
    `Results` are the cumulative totals from all the random points
    generated.

    :   **Parameters:**: `totalPoints` - the total number of random points to compute. **Returns:**: the last random interior point of the convex polytope computed by this method.

---


### run

```
public CdHarComputer.Results run(java.util.Map<? extends V,java.lang.Double> startPoint)
```

:   Computes and returns a random interior point of the convex polytope
    starting from the provided point.

    :   **Parameters:**: `startPoint` - variables mapped to their numerical values. **Returns:**: a random interior point of the convex polytope. **Throws:**: `InvalidPointException` - if the provided point does not lie within the polytope. **See Also:**: `setPoint(Map)`


---


|  |  |  |
| --- | --- | --- |
| **Overview** \| **Package** \| **Class** \| **Use** \| **Tree** \| **Deprecated** \| **Index** \| **Help** | |  |
| PREV CLASS   **NEXT CLASS** | **FRAMES**    **NO FRAMES**     **All Classes** |  |
| SUMMARY: NESTED \| FIELD \| CONSTR \| METHOD | DETAIL: FIELD \| CONSTR \| METHOD |  |


---
